# Centrifugation is an effective and inexpensive way to determine *Batrachochytrium dendrobatidis* quantity in water samples with low turbidity

**DOI:** 10.1007/s00442-024-05604-0

**Published:** 2024-08-14

**Authors:** Taegan A. McMahon, Tatum S. Katz, Kate M. Barnett, Bridget A. Hilgendorff

**Affiliations:** 1 Department of Biology, Connecticut College, New London, CT 06320 USA; 2 Ecology, Evolution, and Marine Biology Department, University of California Santa Barbara, Santa Barbara, CA 93106 USA; 3 Present Address: U.S. Department of Agriculture, Agricultural Research Service, Roman L. Hruska U.S. Meat Animal Research Center, Clay Center, NE USA; 4 Department of Biology, Emory University, Atlanta, GA 30322 USA

**Keywords:** Environmental detection, Chytridiomycosis, Chytrid fungus, Bd quantification, EDNA, Chytrid, Amphibian, Extraction, Environmental detection

## Abstract

*Batrachochytrium dendrobatidis* (Bd) is a pathogenic chytrid fungus that is particularly lethal for amphibians. Bd can extirpate amphibian populations within a few weeks and remain in water in the absence of amphibian hosts. Most efforts to determine Bd presence and quantity in the field have focused on sampling hosts, but these data do not give us a direct reflection of the amount of Bd in the water, which are useful for parameterizing disease models, and are not effective when hosts are absent or difficult to sample. Current methods for screening Bd presence and quantity in water are time, resource, and money intensive. Here, we developed a streamlined method for detecting Bd in water with low turbidity (e.g., water samples from laboratory experiments and relatively clear pond water from a natural lentic system). We centrifuged water samples with known amounts of Bd to form a pellet and extracted the DNA from that pellet. This method was highly effective and the resulting concentrations across all tested treatments presented a highly linear relationship with the expected values. While the experimentally derived values were lower than the inoculation doses, the values were highly correlated and a conversion factor allows us to extrapolate the actual Bd concentration. This centrifuge-based method is effective, repeatable, and would greatly expand the domain of tractable questions to be explored in the field of Bd ecology. Importantly, this method increases equity in the field, because it is time- and cost-efficient and requires few resources.

**Supplementary Information:**

The online version contains supplementary material available at 10.1007/s00442-024-05604-0.

## Introduction

*Batrachochytrium dendrobatidis* (Bd) is a pathogenic fungus that is particularly lethal for amphibians (Scheele et al. 2019; Stuart et al. 2004) and can be lethal in crayfish at high concentrations (McMahon et al. 2013; Nordheim et al. 2021). Bd has been linked to amphibian population extirpation and species extinctions (Scheele et al. 2019; Stuart et al. 2004), and it can persist even after amphibian extirpation, because it appears to have non-amphibian hosts (Brannelly et al. 2015; McMahon et al. 2013; Oficialdegui et al. 2019) and can remain viable in the absence of amphibians (Johnson and Speare 2003; Rumschlag et al. 2021).

Currently, most methods used to quantify Bd infection in pond systems and laboratory experiments rely on screening host tissue samples and skin swabs. While swabbing hosts for Bd is effective, it is not a practical surveillance tool when hosts cannot be easily obtained (e.g., endangered, dormant, or cryptic species). Bd has been shown to persist environmentally for short periods of time in the absence of known hosts (Pilliod et al. 2014; Rumschlag et al. 2021), which has likely made reestablishment efforts of susceptible species difficult (e.g., Stockwell et al. 2008). Researchers have used water filtration (Hyman and Collins 2012; Kirshtein et al. 2007) and eDNA detection (Brannelly et al. 2020; Chestnut et al. 2014; Kamoroff and Goldberg 2017) to screen water samples for Bd DNA. These methods are effective, but are generally limited as they are expensive, time-consuming, and require specialized skills and training.

Researchers also use destructive or invasive sampling methods to screen aquatic organisms for Bd, for example, tadpole mouthpart swabbing or extraction, and GI tract extraction in freshwater invertebrates (Brannelly et al. 2015; Hyatt et al. 2007; McMahon et al. 2013; McMahon and Rohr 2015). Animal-based sampling methods such as tadpole mouthpart swabbing or lethal sampling eliminate the possibility of repeated measurements from the same individual due to destruction or animal welfare concerns and increase the sample sizes required for experiments with multiple time points, further burdening freshwater taxa populations. Additional non-invasive, easily repeatable methods would also enable us to more safely study vulnerable host populations. Importantly, while screening hosts gives us important prevalence and intensity information, infection intensity and pathogen shedding can have a nonlinear relationship (McCallum et al. 2017), and it is hypothesized that the majority of Bd transmission does not occur due to physical contact among hosts but instead through contact with the aquatic infectious zoospore stage (Rachowicz and Briggs 2007; Kilpatrick et al. 2010). Therefore, screening a host for infection is not the best method for estimating transmission parameters; instead, quantifying the rate at which an infected host sheds zoospores into a waterbody is more appropriate.

Parameterizing disease models with precise laboratory data is becoming increasingly important as we develop a stronger understanding of the wide-ranging impact of emerging pathogens. Given the high levels of variation inherent in disease systems, model parameterization requires high sample sizes for accuracy, making laboratory experiments a critical tool to reinforce model development. Disease models provide us with predictive frameworks that can be utilized for both established pathogen outbreaks and with new emerging diseases (e.g., see Briggs et al. 2010; Wilber et al. 2021). Determining the shedding rate of an infectious host, for example, helps us understand and predict disease risk at a population and community level. Modeling pathogen transmission aids the design and evaluation of disease mitigation strategies, which is incredibly valuable for optimizing management strategies for conservation crises (Barnett and Civitello 2020).

As noted above, researchers have developed effective eDNA screening methods (Brannelly et al. 2020; Hyman and Collins 2012; Kirshtein et al. 2007), but they all utilize expensive filters (Table 1). In some cases, these methods have been found to be incredibly valuable detection tools, e.g., with highly turbid water samples, but they can be cost prohibitive. This is an incredibly important consideration, especially given that many early stage researchers may not have access to large grants for funding. Other researchers have identified that these costly methods can be inaccessible and have sought to develop more cost-effective eDNA methods for Bd, yet even their improved method still requires the use of filters (González et al. 2021). Here, we studied an effective, quick, and relatively inexpensive (Table 1) method for screening water samples with low turbidity for Bd presence and abundance. The method we describe below increases ease and accessibility of assessing actual disease risk by determining zoospore shedding rates or quantifying Bd loads in natural lentic systems. While this screening method may not be effective or appropriate for all sampling scenarios, we offer it as an alternative when other eDNA methods are resource prohibitive and suggest that it is especially useful for laboratory experiments where sample volume and turbidity are relatively low. Importantly, methods that reduce time and cost burdens increase equity in science, which is an important consideration for methodological development.

**Table 1:** Cost comparison of consumables needed for the current best eDNA filtration method for *Batrachochytrium dendrobatidis *DNA (Brannelly et al. 2020) vs the centrifugation method described here

	Centrifugation Method					eDNA Filtration Method			
Item	Pack cost	Manufacturer	Product number	Cost per reaction	Item	Pack cost	Manufacturer	Product number	Cost per reaction
2 eppendorf tubes	500 for $66.20	Sigma- Aldrich	T9661	$0.27	0.45-µm microfunnel filter unit	50 for $304.50	Pall Inc.	4800	$6.09
3 pipette tips	1000 for $24.20	Millipore Sigma	Z740030	$0.07	soil prep kit, 1 reaction	50 reactions for $454.50	Qiagen	47014	$9.09
40 ul prepman ultra	200 reactions for $176	Applied Biosystems	4318930	$0.88	12 pipette tips	1000 for $24.20	Millipore Sigma	Z740030	$0.29
	Total Cost of Consumables			$1.22		Total Cost of Consumables			$15.47
			Additional Equipment Required						
		Centrifuge					Centrifuge		
		Heat block				Vortex and adapter			
		Bead beater					Vacuum pump		

Additionally, listed are the equipment required for DNA extraction that a lab will likely already possess, therefore cost information is not included. Price information was collected on 12/28/2023

## Materials and methods

The methods for this experimental work were conducted twice in two independent labs (hereafter, Lab 1 and Lab 2) to determine reliability and replicability across labs. Both labs utilized the same methods unless specifically noted (Table S1).

For clarity, we describe an overview of the methods, and then, we provide more detail below for specific methodology. We created Bd + stocks across a range of concentrations and treated the stocks in several ways: (1) immediate DNA extraction (immediate processing); (2) freezing then DNA extraction (frozen); (3) heat-killing then DNA extraction (heat-killed); and (4) immediate DNA extraction, but using pond water instead of artificial spring water (ASW; Cohen et al. 1980; immediate processing–pond water) to trial a variety of situations that may be encountered in both the field and lab. Lab 1 performed the heat-killed and pond water treatments, while Lab 2 performed the immediate processing, heat-killed, and freezing treatments. With the replicates that were processed immediately, a variety of centrifugation protocols were trialed. Additionally, we created stocks that contained zoospores only or both zoospores and zoosporangia across a range of concentrations to determine if there was an impact of life stage on our findings.

### Bd culture and Bd + Stock

Bd [California Sierran strains: JEL 270, CJB4, CJB5-(2), CJB7, TST77] was grown on 1% tryptone plates for 5–14 days. The zoospores and zoosporangia were harvested from each plate by flooding the plates with sterile ASW or sterile distilled water. The liquid suspension from all plates was collected to create a Bd + stock, which contained both zoospores and zoosporangia. The concentrations of all stocks were determined using a hemocytometer. These stocks were individually serially diluted to create a range of concentrations (10, 100, 1000, 10,000, and 100,000 zoospores/ml) using either sterile water (immediate preparation, frozen, heat-killed) or pond water (immediate preparation–pond water). There was also a Bd-negative (Bd-) control to verify that there was no contamination and that the quantitative PCR (qPCR) ran correctly; contamination was not a problem and the Bd- replicates were Bd free. After dosing, the vials were inverted 12 times to fully mix the solution.

### Zoospore-only stock

To create a zoospore-only stock, we took part of the Bd + stock, which contained zoospores and zoosporangia, and passed it through a 5 µm filter to remove the zoosporangia. A visual inspection (*n* = 4) was conducted with a hemocytometer to verify that all zoosporangia were filtered out. The concentrations of the zoospore-only stock were also determined using a hemocytometer following filtration (actual concentrations were 9, 90, 900, 9000, and 90,000 zoospores/ml; *n* = 4 replicates/concentration). The zoospore-only stock had a lower concentration after filtration, because some zoospores were lost during the filtration process.

### Preparation of treatments

To determine if there is an effect of sample storage method on protocol accuracy, we exposed the Bd + stock (zoospores and zoosporangia) to several treatments. We wanted to determine if it was more effective to process the samples immediately (i.e., alive), if they could be frozen first (to increase convenience of the methodology, because samples could be collected, preserved, and processed at a later date), or if they should be processed immediately but killed first using a heat-killing process (potentially needed to ensure sufficient Bd pelleting). Furthermore, we also included a treatment where stocks were prepared with low-turbidity pond water (turbidity of 3 NTU, collected from New London, CT) instead of sterile water to see if these methods could extend to relatively clear water found in natural systems. The samples that were frozen were stored overnight in a laboratory grade – 20 °C freezer, and then thawed prior to centrifugation (*n* = 3 per concentration). The samples that were heat-killed and then centrifuged (*n* = 3 per concentration) were placed in a dry heat block at 40 °C for 1 h prior to centrifugation. The heat-killed samples were allowed to cool briefly prior to centrifugation to avoid bursting the tubes.

### Centrifugation testing

Not all labs have access to a centrifuge that will reach high RPM speeds, and developing methods that are equitable across labs is important, so we centrifuged the samples that were immediately processed in one of two ways: a) 13,000 RPM for 5 min (*n* = 36); or b) 6,000 RPM for 8 min (*n* = 54). Lab 1 used the 13,000 RPM for 5 min protocol on 15 ml volumes, and Lab 2 used the 6000 RPM for 8 min protocol on 1.5 ml volumes. A 2000 RPM for 10-min protocol was also tested; however, no pellet/smear was seen and all samples were qPCR negative for Bd; therefore, this protocol was abandoned. After all samples were centrifuged, we carefully removed the liquid in suspension above the Bd pellet/smear at the bottom of the tube (see Figure S1).

### Bd DNA extraction and qPCR

Immediately following centrifugation, DNA extraction was completed within the same microcentrifuge tube to reduce sample loss. We used PrepMan Ultra (Applied Biosystems) to extract DNA from all samples. ﻿Forty μl of PrepMan Ultra was added to each tube and the tubes were agitated in a bead beater for 45 s and then centrifuged for 30 s at 13,000 rpm; tubes were bead beaten and centrifuged three times in succession. Following bead beating and centrifugation, tubes were placed in a heat block at 100 °C for 10 min, allowed to cool for 2 min, and centrifuged again at 13,000 rpm for 3 min. Supernatant from the final centrifugation contains DNA and was used for qPCR.

We processed all samples to determine Bd zoospore genome equivalents (GE, Lab 1) or zoospore equivalents (ZE, Lab 2) using the qPCR protocol described by Hyatt et al (2007). For each qPCR plate, we used two (Lab 1) or three (Lab 2) negative controls, plasmid-based (Lab 1, 3 replicates each of standards ranging from 0.84-840GE, Pisces Molecular) or zoospore-based Bd standards (Lab 2, 3 replicates each of 0.1ZE, 1ZE, 10ZE, and 100ZE), and TaqMan Exogenous Internal Positive Control Reagents (Applied Biosystems) to screen for inhibition; there was no evidence of inhibition. Reactions were run in singlicate. After determining the GE for each sample, we converted these values to zoospore equivalents by multiplying the genomic equivalents by a factor of 3.16 (determine by regressing each lab’s standard curves against one another), and then, we compared the expected number of zoospores to the observed number of zoospores across all samples.

### Statistical analyses

All analyses were conducted using R version 4.0.3 (R Development Core Team 2020). We ran an Analysis of Covariance (package: jmv, function: ANCOVA) to test the effect of lab (including differences in personnel, centrifuge times, and standards), stock preparation (zoospores and zoosporangia or zoospores only), or preparation treatment (immediately processed, frozen, heat-killed, or immediately processed–pond water) on observed vs. expected zoospore concentration. Further, Spearman’s Rank (package: stats, function: cor.test) correlation tests were run for preparation treatment groups to compare expected (dosed) Bd concentrations with observed zoospore equivalent qPCR output. All tests were run at an alpha level of 0.05.

## Results

**Fig. 1:**
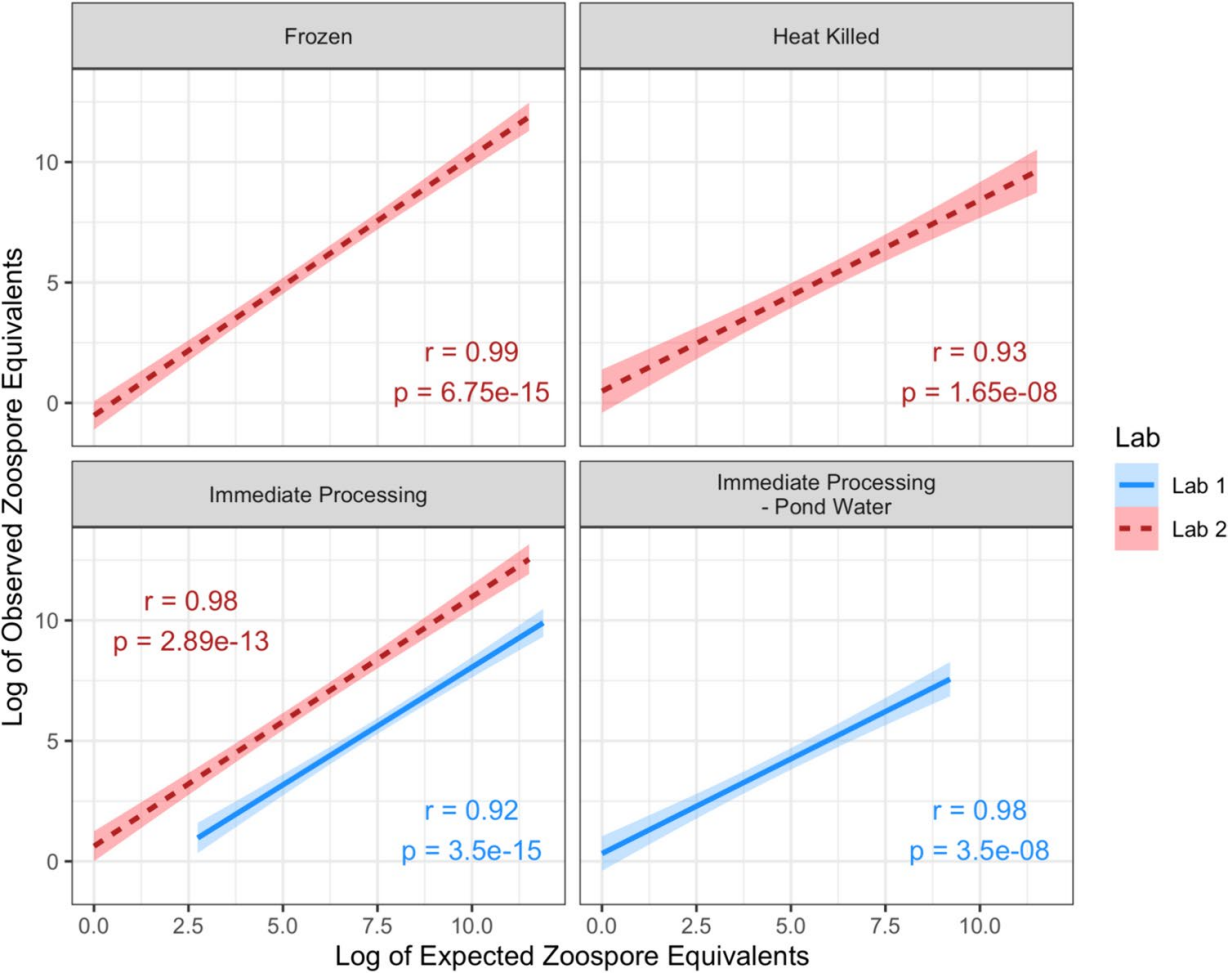
Observed vs. expected *Batrachochytrium dendrobatidis* zoospore equivalents across stock preparation treatments (all treatments used sterile distilled water except the pond water treatment). Results for zoospores only and zoospores and zoosporangia are presented together, because there was no difference observed between these groups. Experiments were conducted in two labs to assess consistency of the protocol, and these data are presented here, separately. Sample sizes were as follows: Frozen (*n* = 3), Immediate processing (*n* = 3), Heat killed (*n* = 8 for Lab 1, and *n* = 4 for Lab 2), and Immediate processing–Pond water (*n* = 3). *R* and *p* values presented are from Spearman’s rank tests; bands represent 95% confidence intervals

There was an effect of lab in determining the observed zoospore concentration given the expected zoospore concentration (ANCOVA, *p* < 0.0001); however, both labs independently achieved very strong and significant correlations between the observed and expected zoospore equivalents (Spearman’s rank, all $\rho$ > 0.90, all p values < 0.05, Fig. 1). There was no effect of whether zoospores were filtered out of the stock or not in determining the observed zoospore concentration (ANCOVA, *p* = 0.22). Additionally, there was a significant negative effect of the heat-killing treatment preparation (ANCOVA, *p* = 0.0014) in determining the observed zoospore concentration, while freezing or pond water had no effect (ANCOVA, all p values > 0.05). Regardless of preparation treatment, all observed zoospore equivalent values were significantly strongly correlated to the expected concentrations (Spearman’s rank, all $\rho$ > 0.90, all *p* values < 0.05, Fig. 1).

## Discussion

Here, we present a set of simple centrifugation methodologies that yield accurate estimates of Bd in small volumes of low-turbidity water. These methods require a centrifuge that reaches at minimum 6,000 RPM but do not require any additional major equipment that is not already used during the average qPCR protocol (e.g., see Hyatt et al. 2007). Previous methodologies require expensive filters (i.e., Brannelly et al. 2020, 0.45 μm MicroFunnel Filter, Pall Corporation, Port Washington, New York; currently available for $6.09 per unit, Table 1), which is often cost prohibitive given that each sample requires a new filter. The methods described here provide an effective, cost-efficient, and more accessible and equitable path forward for screening low-turbidity water for Bd, as many researchers (especially graduate students, early career researchers, and conservation agencies) do not always have access to large sources of funding.

We determined that all of the protocols yielded reasonably accurate estimates of Bd concentration (all $\rho$ > 0.90, all *p* values < 0.05) in the water, except the 2000 RPM for 10 min protocol, which did not yield pellets/smears and gave negative qPCR reads regardless of Bd concentration. Any of the other protocols described above could be utilized to provide accurate estimates of Bd concentration in water; therefore, a lab could use whichever protocol they need depending on the type of centrifuge they have access to.

In addition, while we found that samples can be processed immediately upon collection or frozen prior to processing, we do not recommend heat-killing the fungus prior to processing. While heat-killing the fungus still yielded relatively accurate results (Fig. 1), they were not as accurate as processing immediately or freezing the samples first. Therefore, we suggest researchers not heat-kill their samples unless necessary for their own protocol as it provides no benefit and instead lowers accuracy of Bd concentration estimates. Freezing the samples, however, greatly increases the convenience and feasibility of these methods as it allows the samples to be collected on one day and processed on another without risking degradation of Bd DNA.

Notably, we ran these experiments in two different labs using the same protocol to test repeatability. While there was a significant difference between labs for quantifying dosed Bd, both labs independently achieved strong and significant correlations in their observed and expected doses. Therefore, these methods are repeatable across labs regardless of which centrifuge time, strain, or type of standard is used. We suggest that each lab independently validates their own standard curve for these methods to ensure that other lab-based differences do not affect accuracy of results (see Supplementary Material for validation protocol).

While effective, our methodologies are limited by centrifugation tube size, and may be limited by water turbidity as well. We screened relatively clear pond water with low turbidity in our experiments and acknowledge that not all field systems have clear water. The use of previously established filter methodologies may be more ideal in those scenarios (see Brannelly et al. 2020), but more research is needed to determine how effective this new centrifugation methodology might be with sediment-filled water. Furthermore, this study was conducted in 1.5 ml and 15 ml centrifuge tubes, on relatively small volumes of water. However, it is important to note that this sample size is sufficient for lab experiments designed to quantify shedding rates for model parameterization, a highly important use case for this method. Protocols requiring larger volumes of water may consider centrifuging larger volumes of water (as allowed by centrifuge size), or pooling replicate samples at any point in the procedure. Otherwise, samples of large volume may be better suited for processing using filtration eDNA methods. Finally, researchers collecting eDNA samples in the field should consider which method is best given logistical constraints. On-site filtration methods may be time-consuming, but the samples could be easier to transport and permissions may be more easily granted; meanwhile, transporting water samples could save time in the field and allow for the collection from a greater number of sites, but may require different permissions.

Bd has devastated hundreds of species and impacted far more globally (Scheele et al. 2019; Stuart et al. 2004). Given that this conservation crisis is ongoing, innovation and the development of new methodologies that allow us to address previously unanswerable questions are of considerable significance. It is not only important that these new methods are effective but also that they increase equity and access to information across the scientific field. Our centrifugation methods are more cost-effective compared to previous methods and do not utilize additional expensive materials or equipment. Considering consumables only, centrifugation-based methods are approximately 33 times less expensive than established filtration-based methods (Brannelly et al. 2020, comparison calculations based off current consumable pricing). This is significant, because financial barriers have a massive and disproportionate impact on underrepresented researchers and science. As a community, researchers must continue to develop new, equity-minded methodologies that strengthen our knowledge and community.

### Supplementary Information

Below is the link to the electronic supplementary material.

Supplementary file1 (DOCX 6510 KB)

## Data Availability

Data and analysis code are available via Dryad Digital Repository.

## Declarations

### Benefit-sharing section

This research provides low-cost eDNA methods that can be utilized by all researchers, including those without large grant fund access, increasing equity in the field. These methods will be useful in advancing our understanding of chytridiomycosis, a disease posing a dire conservation threat to hundreds of amphibian species. All collaborators on this work are listed as co-authors.
