# Supplementary material for: Centrifugation is an effective and inexpensive way to determine Batrachochytrium dendrobatidis quantity in water samples with low turbidity
Source: Oecologia. 2024 Aug 14;205(3-4):437–43. doi: 10.1007/s00442-024-05604-0 (PMC11358168; doi:10.1007/s00442-024-05604-0)
Supplement: Supplementary file 1 — Supplementary file1 (DOCX 6510 KB) [file 442_2024_5604_MOESM1_ESM.docx]

Electronic Supplementary Material

Title: Centrifugation is an effective and inexpensive way to determine *Batrachochytrium dendrobatidis* quantity in water samples with low turbidity

Taegan A McMahon^1*^, Tatum S Katz^23^ , Kate M Barnett^4^, Bridget A Hilgendorff^1^

^1^Connecticut College, Department of Biology, New London, Connecticut 06320, USA

^2^University of California Santa Barbara, Ecology, Evolution, and Marine Biology Department, Santa Barbara, California 93106, USA

^3^ Current Address: U.S. Department of Agriculture, Agricultural Research Service, Roman L. Hruska U.S. Meat Animal Research Center, Nebraska, USA

^4^Emory University, Department of Biology, Atlanta, Georgia 30322, USA

*Corresponding author: Taegan McMahon, [taeganmcmahon@gmail.com](mailto:taeganmcmahon@gmail.com)

Electronic Supplemental Materials

**Supplementary Information Guide**

Supplemental Tables and Figures

Supplemental Protocol

Supplemental Tables

Figure S1. *Batrachochytrium dendrobatidis* (Bd) precipitate or pellet formed at the bottom of a water sample that has been centrifuged at 13,000 RPM for 5 minutes.

Supplemental Protocol
